# Recommended homemade fluid utilization for the treatment of diarrhea and associated factors among children under five in sub-Saharan African countries: a multilevel analysis of the recent demographic and health survey

**DOI:** 10.1186/s12887-024-04810-2

**Published:** 2024-05-10

**Authors:** Belayneh Shetie Workneh, Enyew Getaneh Mekonen, Mohammed Seid Ali, Almaz Tefera Gonete, Masresha Asmare Techane, Mulugeta Wassie, Alemneh Tadesse Kassie, Medina Abdela Ahmed, Sintayehu Simie Tsega, Yilkal Abebaw Wassie, Alebachew Ferede Zegeye, Berhan Tekeba, Tadesse Tarik Tamir

**Affiliations:** 1 Department of Emergency and Critical Care Nursing, School of Nursing, College of Medicine and Health Sciences, University of Gondar, Gondar, Ethiopia; 2 Department of Surgical Nursing, School of Nursing, College of Medicine and Health Sciences, University of Gondar, Gondar, Ethiopia; 3 Department of Pediatrics and Child Health Nursing, School of Nursing, College of Medicine and Health Sciences, University of Gondar, Gondar, Ethiopia; 4 School of Nursing, College of Medicine and Health Sciences, University of Gondar, Gondar, Ethiopia; 5 Department of Clinical Midwifery, School of Midwifery, College of Medicine and Health Sciences, University of Gondar, Gondar, Ethiopia; 6 Department of Medical Nursing, School of Nursing, College of Medicine and Health Sciences, University of Gondar, Gondar, Ethiopia

**Keywords:** Children under-five, Homemade fluid, Diarrhea, Sub-saharan Africa, Utilization

## Abstract

**Introduction:**

Diarrhea is a common public health problem and the third leading cause of death in the world among children under the age of five years. An estimated 2 billion cases and 1.9 million deaths are recorded among children under the age of five years every year. It causes body fluid loss and electrolyte imbalance. Even though, early initiation of recommended homemade fluid is a simple and effective approach to prevent diarrhea-related complications and mortality of children, recommended homemade fluid utilization for the treatment of diarrhea is still low in sub-Saharan African countries. Therefore, this study aimed to assess the magnitude of recommended homemade fluid utilization for the treatment of diarrhea and associated factors among children under five in sub-Saharan African countries.

**Method:**

The most recent Demographic and Health Survey dataset of 21 sub-Saharan African countries from 2015 to 2022 was used for data analysis. A total of 33,341 participants were included in this study as a weighted sample. Associated factors were determined using a multilevel mixed-effects logistic regression model. Significant factors in the multilevel mixed-effect logistic regression model were declared significant at *p*-values < 0.05. The adjusted odds ratio (AOR) and confidence interval (CI) were used to interpret the results.

**Result:**

The overall recommended homemade fluid utilization for the treatment of diarrhea among children under five in sub-Saharan African countries was 19.08% (95% CI = 18.66, 19.51), which ranged from 4.34% in Burundi to 72.53% in South Africa. In the multivariable analysis, being an educated mother/caregiver (primary and secondary level) (AOR = 1.15, 95% CI: 1.04, 1.27) and (AOR = 1.30, 95% CI: 1.15, 1.1.47), the primary and secondary level of fathers education (AOR = 1.53, 95% CI: 1.37, 1.71) and (AOR = 1.41, 95% CI: 1.19, 1.1.68), having antenatal care follow-up (AOR = 1.16, 95% CI: 1.01, 1.33), having multiple children (AOR = 1.17, 95% CI: 1.07, 1.28), and being an urban dweller (AOR = 1.15, 95% CI: 1.04, 1.27) were factors associated with recommended homemade fluid utilization.

**Conclusion:**

The overall recommended homemade fluid utilization for the treatment of diarrhea was low. Individual and community-level variables were associated with recommended homemade fluid utilization for the treatment of diarrhea. Therefore, special consideration should be given to rural dwellers and caregivers who have three and below children. Furthermore, better to strengthen the antenatal care service, mother/caregiver education, and father’s education to enhance recommended homemade fluid utilization for the treatment of diarrhea.

## Introduction

According to the World Health Organization (WHO) definition, diarrhea is the passage of three or more loose or liquid stools per day (more frequent passage than normal for the individual). It is a major public health problem and the third leading cause of mortality in children [1]. Based on recent evidence, 2 billion diarrheal diseases and 1.9 million deaths were reported among children under the age of five years every year globally [2]. Diarrhea disproportionally affects low and middle-income countries due to the limited access to safe water for drinking and cooking, sanitation and hygiene, improper feeding practices, and poor housing sanitation [3, 4]. The result of the study in 34 sub-Saharan African countries reported that 15.3% of children under five had diarrhea [5].

Diarrhea causes body fluid loss and electrolyte imbalance [6]. Replacing fluid with oral rehydration solutions (ORS), a solution of clean water, sugar, and salt is the primary means of treating diarrhea. Because of difficulties in making the oral rehydration solution (ORS) packet available in each home, efforts have been directed at utilizing fluids made from ingredients already available at home [1, 7]. When ORS packets are not available, dehydration can be prevented or delayed by administering homemade solutions made of salt and sugar dissolved in safe water, lightly salted rice water, or even plain water [8].

The findings of the prior studies revealed that homemade fluid utilization ranged from 18.7 to 71.7% in sub-Saharan African countries [9–13]. Mothers/caregivers educational level [10, 12, 14, 15], mothers/caregivers age [10], marital status [10], media exposure [10, 12], working status [10, 12], household wealth [12], distance from health institution [14], and husband educational level [14] were factors significantly associated with recommended homemade fluid utilization for the treatment of diarrhea.

Even though, early initiation of recommended homemade fluid is a simple and effective approach to prevent diarrhea-related complications and mortality of children, recommended homemade fluid utilization for the treatment of diarrhea is still low in sub-Saharan African countries. Therefore, this study aimed to assess the magnitude of recommended homemade fluid utilization for the treatment of diarrhea and associated factors among children under five in sub-Saharan African countries.

## Method

### Data source, study setting, period, and design

A cross-sectional study was conducted. The study used the recent and appended demographic and health survey dataset from 21 sub-Saharan African countries conducted from 2015 to 2022 to carry out a multilevel mixed-effect analysis. DHS is a community-based nationally representative cross-sectional study conducted every five years to examine health and health-related indicators. All children under the age of five who had diarrhea two weeks preceding the survey were included in the analysis.

### Study population, and sampling technique

The most recent (from 2015 to 2022) data set of 21 sub-Saharan African countries (Ethiopia, Guinea, Angola, Benin, Cameron, Burundi, Kenya, Senegal, Gambia, Gabon, Malawi, Liberia, Mali, Sera Leone, Nigeria, Rwanda, Zimbabwe, South Africa, Tanzania, Uganda, and Zambia) was downloaded from the Demographic Health Survey (DHS) program website and appended to have a single data set. DHS data exhibit nested dependencies, where individuals are nested within communities. It employs stratified two-stage cluster sampling. Clusters (communities) are sampled, and within each cluster, households and individuals are further selected. The weighted total sample participants for the study were 33,341 (Table 1).

**Table 1:** Sample size for recommended homemade fluid utilization for the treatment of diarrhea and associated factors among children under five in sub-Saharan African countries

Country	Year of survey	Weighted sample(*n*)	Weighted sample (%)
Angola	2015	1973	5.92
Benin	2017/18	1320	3.96
Burundi	2016/17	2877	8.63
Cameron	2018	1127	3.38
Ethiopia	2016	1227	3.68
Gabon	2021	974	2.92
Gambia	2019/20	1403	4.21
Guinea	2018	1043	3.13
Kenya	2022	2416	7.25
Liberia	2019/20	763	2.29
Mali	2018	1631	4.89
Malawi	2015/16	3584	10.75
Nigeria	2016	3950	11.85
Rwanda	2019/20	1141	3.42
Sera Leon	2019	630	1.89
Senegal	2019	726	2.18
Tanzania	2022	932	2.80
Uganda	2016	2832	8.49
South Africa	2016	356	1.07
Zambia	2018	1422	4.27
Zimbabwe	2015	1014	3.04
Total		33,341	

### Study variables

#### Dependent variable

The dependent variable of this study was recommended homemade fluid utilization for the treatment of diarrhea. The respondents of the study were asked whether the children with diarrhea were given Recommended homemade fluids or not and recorded as “Yes” if children with diarrhea were given recommended homemade fluid during the diarrhea and “No” if children with diarrhea were not given recommended homemade fluid during the diarrhea in KR file data.

#### Independent variables

For assessing factors associated with both individual and community level variables were incorporated.

##### Individual level variables

The age of the mother/caregiver (15–19 years, 20–35 years, and 36–49 years), educational level of the mother/caregiver (no education, primary, secondary, and higher level), having media exposure (yes or no), household wealth index (poor, middle and rich), mother/caregivers working status (working or not working), a fathers education level (no education, primary, secondary and higher level), place of delivery (at home, and health institution), distance from health institution (big problem, and not big problem), number of children (≤ 3 children, and ≥ 4 children), and gender for a household head (female, and male).

##### Community level variables

Residence (urban, and rural), community media exposure (low, and high), community illiteracy (low, and high), community poverty (low, and high), and community level ANC utilization (low, and high).

The community media exposure, community poverty, community illiteracy, and community ANC utilization levels were aggregated from the individual level variables; media exposure (derived from combining whether a respondent reads a newspaper, watches television, and listens to radio and coded as yes (if the respondent had been exposed to at least for one of these media) an no (otherwise), house wealth status (wealth index), maternal educational status, and maternal ANC utilization status. Regarding the analysis of the aggregation, first, the individual variables were re-categorized and cross-tabulations were done with the cluster variable using STATA version 14. Then, the proportion of media exposure, poverty, illiteracy, and ANC utilization was computed using Microsoft Excel 2013. Next, the proportions from Excel were imported to STATA and combined with the original set of the variables in the STATA. Finally, we have categorized the proportion of media exposure, poverty, illiteracy, and ANC utilization into levels [16].

### Statistical analysis

The cleaned and recoded data were analyzed using STATA (version 14) statistical software. Missing data for the outcome variable were dropped. The weighting sample (v005/1,000,000) was applied to address issues related to under or over-sampling. Variance inflation factor (VIF) was tested to check multi-collinearity between variables with the findings ranging from 1 to 2.20 and a mean-variance inflation factor of 1.45. To determine factors associated with the outcome variable multi-level mixed-effect logistic regression was applied. Four models model I, model II, model III, and model IV were used to assess the variability of recommended homemade fluid utilization for the treatment of diarrhea across the cluster, the association of individual-level variables with the outcome variable, the association of community-level variables with the outcome variable, and association of both individual and community variables with outcome variable respectively. Variables with a *p*-value of < 0.25, were candidates for the multivariable analysis in univariate analysis at 95% confidence intervals and variables with a *p*-value of ≤ 0.05 were considered as significantly associated with the outcome variable in the final analysis.

### Ethical consideration

Since we have used secondary (publicly accessible) data, obtaining ethical approval for the study was not needed. However, by registering (online requesting) we have accessed the data set from http://www.dhsprogram.com.

## Results

### Socio-demographic characteristics of the study subjects in terms of recommended homemade fluid utilization

More than two-thirds 24,174 (72.50%) of the mothers were in the age category of 20–35 years. Two-thirds of 22,102 (66.29%) mothers had media exposure. Nearly two-thirds of 21,642 (64.91%) of the mothers had work. More than half 18,748 (59.27%) of the mothers faced big problems in accessing the health institution. The majority 25,191 (90.91%) of the mothers had ANC follow-up. More than two-thirds of 22,737 (68.20%) of the study subjects were rural dwellers (Table 2).

**Table 2:** The distribution of recommended homemade fluid utilization for the treatment of diarrhea across the independent variables

Individual level variables		Weighted frequency (*n*)	Percentage (%)	Recommended homemade fluid utilization	
				No (%)	Yes (%)
Maternal age	15–19 years	2629	7.88	81.09	18.91
	20–35 years	24,174	72.50	80.92	19.08
	36–49 years	6539	19.61	80.83	19.17
Maternal educational level	No education	11,100	33.29	82.73	17.27
	Primary	12,611	37.82	81.95	18.05
	Secondary	8,408	25.22	77.19	22.81
	Higher	1,223	3.67	79.38	20.62
Media exposure	No	11,238	33.71	82.71	17.29
	Yes	22,102	66.29	80.01	19.99
Household wealth	Poor	15,765	47.28	81.51	18.49
	Middle	6,589	19.76	80.99	19.01
	Rich	10,987	32.95	80.02	19.98
Currently working	No	11,699	35.09	79.94	20.06
	Yes	21,642	64.91	81.45	18.55
Father’s educational level	No education	9,335	32.89	83.16	16.84
	Primary	9,270	32.66	82.93	17.07
	Secondary	7,625	26.86	76.47	23.53
	Higher	2,154	7.59	78.01	21.99
Number of children	≤ 3	20,611	61.82	80.64	19.36
	≥ 4	12,731	38.18	81.37	18.63
Have ANC follow-up	No	2,517	9.09	83.99	16.01
	Yes	25,191	90.91	80.66	19.34
Place of delivery	At home	8,952	26.85	82.92	17.08
	At health institution	24,390	73.15	80.18	19.82
Distance from health institution	Big problem	12,884	40.73	81.55	18.45
	Not big problem	18,748	59.27	81.23	18.77
Gender of household head	Male	187,906	79.07	80.90	19.10
	Female	49,732	20.93	81.00	19.00
Residence	Urban	10,604	31.80	80.14	19.86
	Rural	22,737	68.20	81.28	18.72
Community media exposure	Low	17,509	52.51	81.52	18.48
	High	15,833	47.49	80.25	19.75
Community illiteracy	Low	19,499	58.48	81.54	18.46
	High	13,842	41.52	80.04	19.96
Community poverty	Low	17,194	51.57	81.82	18.18
	High	16,148	48.43	79.96	20.04
Community-level ANC utilization	Low	13,130	39.39	81.06	18.94
	High	20,207	60.61	421.45	19.18

### Recommended homemade fluid utilization for the treatment of diarrhea among children under five in sub-Saharan African countries

**Fig. 1:**
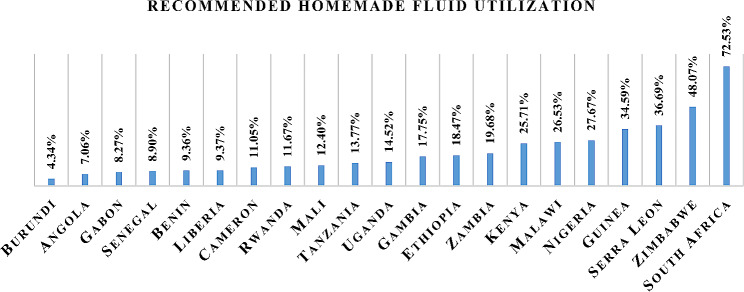
Recommended homemade fluid utilization for the treatment of diarrhea among children under five in sub-Saharan African countries

The overall recommended homemade fluid utilization for the treatment of diarrhea among children under five in sub-Saharan African countries was 19.08% (95% CI 18.66%, 19.51%), which ranged from 4.34% in Burundi to 72.53% in South Africa (Fig. 1).

### Multivariable multilevel logistic regression of recommended homemade fluid utilization for the treatment of diarrhea among children under five in sub-Saharan African countries

In the final fitted model of multivariable logistic regression mother/caregiver educational level, husband/partner educational level, antenatal care follow-up, distance from health institution, and urban residence were factors significantly associated with homemade fluid utilization (Table 3).

The odds of recommended homemade fluid utilization for the treatment of diarrhea are 15% (AOR = 1.15, 95% CI: 1.04, 1.27) and 30% (AOR = 1.30, 95% CI: 1.15, 1.1.47) higher among mother/caregiver who had primary and secondary educational level as compared with those mother/caregiver who had no education respectively. The likelihood of recommended fluid utilization was 17% (AOR = 1.17, 95% CI: 1.07, 1.28) higher among mothers who had four or more children as compared with those mothers who had three and below children. Recommended homemade fluid utilization was 18% (AOR = 1.18, 95% CI: 1.09, 1.27) higher among mothers/caregivers who reside far from health institutions as compared with those mothers/caregivers who reside near the health institutions. The odds of recommended homemade fluid utilization for the treatment of diarrhea were 53% (AOR = 1.53, 95% CI: 1.37, 1.71) and 41% (AOR = 1.41, 95% CI: 1.19, 1.1.68) higher among mother/caregiver whose husband with primary and secondary educational level as compared with those mother/caregiver whose husband had no education. The likelihood of recommended fluid utilization was 16% (AOR = 1.16, 95% CI: 1.01, 1.33) higher among mother who had ANC follow-up as compared with their counterparts. Recommended homemade fluid utilization was 15% (AOR = 1.15, 95% CI: 1.04, 1.27) higher among urban dwellers as compared with rural dwellers. There was about 10.45% variability in recommended homemade fluid utilization due to the difference between communities/clusters as the ICC value showed. The final model (model III), attributed approximately 10.53% of the variation in the likelihood of recommended homemade fluid utilization to both individual and community-level variables. The lowest deviance, which was 20,570.27 in model III revealed that model III was the best fit for the data (Table 3).

**Table 3:** Multivariable multilevel logistic regression analysis of individual-level and community-level factors associated with recommended homemade fluid utilization for the treatment of diarrhea among children under five in sub-Saharan African countries

Parameter	Null model	Model I AOR(95% CI)	Model II AOR(95% CI)	Model III AOR(95% CI)
Have media exposure				
No		1		1
Yes		1.01 (0.93, 1.10)		0.99 (0.92, 1.08)
Maternal age				
15–19 years		1		1
20–35 years		0.95 (0.82, 1.09)		0.91 (0.79, 1.04)
36–49 years		0.99 (0.83, 1.17)		0.90 (0.76, 1.07)
Education level				
No education		1		1
Primary		1.06 (0.96, 1.17)		**1.15 (1.04, 1.27)** ^*^
Secondary		1.20 (1.06, 1.35)		1.30 (1.15, 1.47)*
Higher		0.94 (0.73, 1.20)		0.94 (0.73, 1.21)
Number of children				
≤ 3		1		1
≥ 4		1.10 (1.01, 1.20)		1.17 (1.07, 1.28)*
Household wealth				
Poor		1		1
Middle		1.00 (0.91, 1.10)		1.01 (0.91, 1.11)
Rich		0.98 (0.89, 1.08)		0.94 (0.85, 1.05)
Have work				
No		1		1
Yes		0.98 (0.91, 1.06)		1.03 (0.95, 1.11)
Distance from health institution				
Not big problem		1		1
Big problem		1.09 (1.01, 1.18)		1.18 (1.09, 1.27)*
Father’s education				
No education		1		1
Primary		0.98 (0.89, 1.09)		1.07 (0.96, 1.19)
Secondary		1.40 (1.26, 1.56)		1.53 (1.37, 1.71)*
Higher		1.33 (1.12, 1.58)		1.41 (1.19, 1.68)*
Place of delivery				
At home		1		1
At health institution		1.05 (0.96, 1.15)		1.00 (0.91, 1.10)
ANC				
No		1		1
Yes		1.18 (1.03, 1.35)		1.16 (1.01, 1.33)*
Gender of household head				
Female		0.90 (0.81, 0.99)		0.91 (0.82, 1.01)
Male		1		1
Residence				
Urban			1.18 (1.10, 1.27)	1.15 (1.04, 1.27)*
Rural			1	1
Community-level media exposure				
Low			1	1
High			1.11 (1.00, 1.24)	1.09 (0.96, 1.23)
Community ANC utilization				
Low			1	1
High			0.96 (0.86, 1.07)	0.91 (0.81, 1.03)
Community illiteracy				
Low			1	1
High			1.13 (1.02, 1.27)	1.06 (0.94, 1.20)
Community poverty				
Low			1	1
High			1.09 (0.98, 1.22)	1.08 (0.95, 1.23)
Variance	0.38	0.37	0.34	0.34
ICC	10.45	10.10	9.39	9.44
PCV	Reference	2.63	10.53	10.53
Deviance	31,907.77	21,032.16	31,056.70	20,570.27

AOR, adjusted odds ratio; ICC, intra-class correlation coefficient; PCV, proportional change in variance; ^*^p ≤ 0.05 (significantly associated)

## Discussion

This study aimed to assess recommended homemade fluid utilization for the treatment of diarrhea and associated factors among children under five in sub-Saharan African countries. The overall recommended homemade fluid utilization for the treatment of diarrhea was 19.08% (95% CI = 18.66, 19.51), which ranged from 4.34% in Burundi to 72.53% in South Africa. It implies that recommended homemade fluid utilization for the treatment of diarrhea was still low and a great concern in sub-Saharan African countries. This finding is lower than the study conducted in Ethiopia [10, 13]. The variation might be due to the difference in study period, sample size (this study was based on pooled analysis), and population (since this study considers study participants in sub-Saharan Africa).

Mother/caregiver educational level, father’s educational level, antenatal care follow-up, distance from health institution, and urban residence were factors significantly associated with homemade fluid utilization. The odds of recommended homemade fluid utilization for the treatment of diarrhea were higher for children of mothers/caregivers who had primary and secondary educational levels as compared with those mothers/caregivers who had no education. This finding is supported by the prior studies [12, 13, 15, 17–19]. It could be because education is the best tool to acquire knowledge including the best approach for the management of diarrhea at home. Thus, those mothers/caregivers who had higher education may have a better understanding and utilization of recommended homemade fluid utilization for the treatment of diarrhea to reduce dehydration-related complications and mortality.

The likelihood of recommended homemade fluid utilization was higher among mothers/caregivers who had ANC follow-up as compared with their counterparts. The finding of this study is supported by the previous study [20]. It could be because those mothers/caregivers who had ANC follow-up may have a high chance of getting counseling from health professionals on how to manage childhood illness including diarrhea. Participants who didn’t receive counseling on child feeding have poor child-feeding practices [21].

Father education was another factor significantly associated with recommended homemade fluid utilization. The odds of homemade fluid utilization were higher among mothers/caregivers who had educated husbands as compared with those mothers/caregivers whose husbands had no education. The finding of this study consistent with the previous study [14]. It could be due to the fact, that education enhances spouses’ level of knowledge about childhood illness including childhood diarrhea and management. Thus, spouses may convince the wives/partners to utilize recommended homemade fluids to rehydrate their child during dehydration. Spouses have a role in children’s and women’s health [22].

The study at hand also revealed that being an urban dweller was associated with higher odds of recommended homemade fluid utilization for the treatment of diarrhea. This finding is in agreement with the prior study [23]. The possible reason might be due to high access and media exposure in urban settings. Media has the potential to change health behaviors by delivering health messages [24]. Furthermore, it could be due to the variation in education between urban and rural dwellers. There is a significant education gap between urban and rural areas [25].

Contrary to the previous studies [26, 27], the finding of this study revealed the odds of recommended homemade fluid utilization were higher among mothers/caregivers who had higher children in number as compared with those mothers/caregivers who had lower children in number. It could be because mothers who have multiple children may have better experience in handling children with diarrhea. Thus, they may use the recommended homemade fluid to rehydrate their child during diarrheal episodes. However, the authors of this study recommended further investigation on this issue.

In addition, the odds of recommended homemade fluid utilization was higher among mothers/caregivers who faced big problems accessing the health institution as compared with those who didn’t face problems accessing the health institution. It could be because those individuals who reside far from the health institution cost money and time to access the health institution during diarrheal episodes. Hence, they may favor managing their child at the home level with recommended homemade fluid instead of seeking health institutions.

This study uses nationally representative data from multiple sub-Saharan countries and appropriate statistical analysis which is multilevel analysis. Hence policymakers and the international community can use it as evidence to undertake necessary measures. However, the study has limitations, important factors that could have a big impact on recommended homemade fluid utilization, like behavior, beliefs, and social norms, are not included in the dataset. Additionally, to measure the fluid utilization during childhood diarrhea a social desirability bias may have been present in the mother’s verbal responses. These will hinder our findings from having the intended impact, so further studies should be carried out to explore recommended homemade fluid utilization during diarrheal disease by observing the frequency and type of fluid offered to the children.

## Conclusion

The overall recommended homemade fluid utilization for the treatment of diarrhea was low. Individual and community-level variables were associated with recommended homemade fluid utilization for the treatment of diarrhea. Therefore, special consideration should be given to rural dwellers and caregivers who have three and below children. Furthermore, better to strengthen the ANC service, mother/caregiver education, and father’s education to enhance recommended homemade fluid utilization for the treatment of diarrhea.

## Data Availability

The datasets generated and/or analyzed during the current study are available publicly online at (https://www.dhsprogram.com).
